# Probing conformational and functional states of human hepatocyte growth factor by a panel of monoclonal antibodies

**DOI:** 10.1038/srep33149

**Published:** 2016-09-09

**Authors:** Masataka Umitsu, Katsuya Sakai, Satoshi Ogasawara, Mika K. Kaneko, Ryoko Asaki, Keiko Tamura-Kawakami, Yukinari Kato, Kunio Matsumoto, Junichi Takagi

**Affiliations:** 1 Laboratory of Protein Synthesis and Expression, Institute for Protein Research, Osaka University, Osaka, 565-0871, Japan; 2 Division of Tumor Dynamics and Regulation, Cancer Research Institute, Kanazawa University, Ishikawa, 920-1192, Japan; 3 Department of Regional Innovation, Tohoku University Graduate School of Medicine, Miyagi, 980-8575, Japan

## Abstract

HGF-Met signaling contributes to various biological events by controlling cell migration. Since the abnormal activation of Met receptor causes cancer progression, inhibitors such as neutralizing antibodies are regarded as promising therapeutics. HGF is secreted as a single-chain (sc) precursor and is processed by extracellular proteases to generate disulfide-bonded two-chain (tc) HGF. Although this proteolytic processing of HGF is necessary for its biological activity, exactly how the proteolysis leads to the conversion of HGF to the active form is still unclar due to the lack of structural information. In order to gain insights about this point, we generated 6 antibodies against HGF. All antibodies recognized different epitopes on the native HGF protein and showed distinct effects when tested in a cell-based HGF-Met signaling assay. They included one antibody (t1E4) that strongly blocks Met activation by tcHGF, as well as one antibody (t8E4) exclusively recognizing the active tcHGF but not inactive scHGF. Thus, a panel of anti-HGF antibodies suitable for probing the structural mechanism of HGF activation were obtained.

The HGF-Met signaling has important biological roles in both normal development and cancer progression. HGF, as a ligand, binds to a receptor tyrosine kinase Met at its extracellular region and activates the kinase activity residing in the cytoplasmic domain. This leads to the phosphorylation of a set of tyrosine residues in the Met cytoplasmic region to recruit downstream signaling proteins, altimately controling the cell’s adhesive and migratory behaviors^1^. Since the alteration in the HGF-Met signaling is known to cause cancer progression, there have been intensive efforts to develop therapeutics targeted at this pathway, either as a small compound that inhibit the kinase activity of Met or an antibody that attenuates HGF-Met interaction^2^,^3^.

It is known that the proteolytic processing of HGF is necessary for its capability to activate Met. HGF has a domain organization very similar to plasminogen, a central enzyme in the fibrinolytic system^4^. Both HGF and plasminogen comprise an N-terminal (N) domain followed by four (in HGF) or five (in plasminogen) consecutive kringle (K) domains, and a C-terminal serine protease (SP) domain (Fig. 1A). The SP domain carries a characteristic catalytic triad comprising His, Ser, and Asp in plasminogen that is responsible for its protease activity, but in HGF His and Ser are not conserved, making it enzymatically nonfunctional^4^. Upon the biosynthesis and the signal peptide cleavage, HGF is secreted into the extracellular space as a single-chain (sc) HGF, where it is cleaved by extracellular proteases such as HGF activator^5^ at the linker region between the 4th kringle (K4) and SP. Since this cleavage occurs at Arg494-Val495 bond which is flanked by two disulfide-bonded Cys residues (Cys487 and Cys604), the resulting product is a disulfide-linked two-chain (tc) HGF^6^. Although both scHGF and tcHGF can bind to Met, only tcHGF can activate Met^7^.

Within the HGF primary structure, the segment encompassing the N to K1 domains (called NK1 hereafter) is reported to contain high affinity binding site(s) for Met^8^,^9^. The NK1-Met interaction is believed to be physiologically relevant, because a therapeutic antibody against Met (onartuzumab) competitively inhibits the binding of NK1 fragment to Met^10^. In addition to NK1, there is another Met-binding interface in the SP domain identified by a crystallographic analysis of a complex between the HGF SP and a fragment of Met comprising Sema and PSI domains^11^. The functional importance of this interface is validated by a mutagenesis study^12^, as well as by the fact that the same interface was found in the crystal structure of complex between the HGF homologue MSP and the Met homologue Ron^13^. Although HGF SP alone cannot activate Met^12^, a simultaneous addition of two HGF fragments, one encompassing N to K4 (called NK4 hereafter) and the other comprising SP, cooperatively activates Met^14^, indicating that the two binding segments can be reconstituted into an active ligand in the absence of the chain-connecting disufide bridge. Due to the lack of the structural information about how the two binding sites engage Met receptor in a cooperated fashion, however, the mechanism of Met activation remains elusive.

Another puzzle that surrounds the HGF-Met signaling pathway is the mechanism of proteolytic activation of HGF. In the HGF SP structure, the N-terminal residue Val495 which becomes exposed upon the cleavage of Arg494-Val495 bond in the full-length HGF inserts its side chain into a hydrophobic pocket of SP domain, which in plasminogen plays a crucial role in the formation of enzymatic active site during the conversion to plasmin^15^. However, this segment is not directly involved in the Met binding, although the primary Met-binding interface is located roughly at the same site as the plasmin’s active site. Since there is no structural information available for the HGF SP before cleavage, it is not clear whether the Val insertion causes a local structural change and indirectly assists the formation of the Met binding site. Another potential mechanism of activation is a global conformational change that may take place in the full-length HGF. Gherardi *et al*. reported that the full-length scHGF and tcHGF exhibit very different shape by using electron microscopy and small angle X-ray scattering analyses^16^. Although these analyses resulted in a hypothetical low-resolution model of Met activation by the signaling-competent tcHGF, we still need multiple high resolution structures for each component to gain insights about the structural change(s) caused by the conversion from scHGF to tcHGF.

Monoclonal antibodies are great tools to distinguish not only different proteins but also among different conformers of a protein. For example, antibodies specific for either scHGF or tcHGF can contribute to the understanding of their structural differences when combined with a detailed epitope mapping. Furthermore, such monoclonal antibodies may be used to facilitate the crystallization and structural determination of each HGF state, as has been used in many applications^17^. Here, we attempted to obtain antibodies recognizing native HGF protein in a conformation-dependent fashion, that can be used as functional and/or conformational probes for HGF.

## Result and Discussion

**Figure 1:**
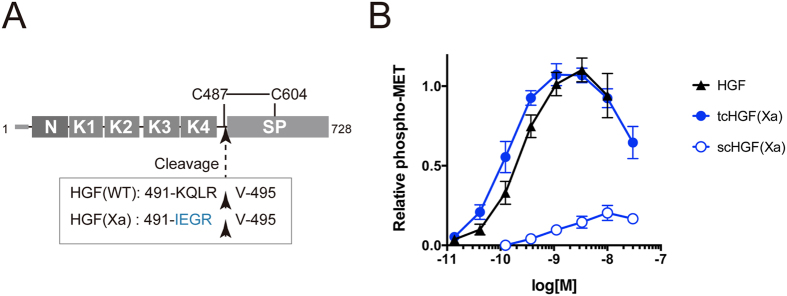
Recombinant HGF protein with the engineered factor Xa site is biologically active. (**A**) A schematic diagram of HGF protein. A disulfied-bond between Cys487 and Cys604^27^ and the 5 amino-acid sequences in the protease-cleavage sites of wild-type and engineered HGF proteins are indicated. A free cystein residue, Cys561, was mutated to serine (C561S) in some of the recombinant proteins used in this study. (**B**) Cellular Met activation by HGF, tcHGF(Xa), and scHGF(Xa). EHMES-1 cells were stimulated with indicated concentrations of recombinant HGF protein for 10 min. The cells were fixed and Met activation was detected by anti-phospho-Met (Tyr1234/1235) antibody. The activities were expressed as a relative Met phosphorylation calculated as described in the Method. Data are mean ± SD of six (HGF and tcHGF(Xa)) or four (scHGF(Xa)) independent experiments.

In order to raise antibodies specifically recognizing either scHGF or tcHGF protein, we decided to use separate antigens for the immunization and the screening. Since the wild-type HGF undergoes spontaneous cleavage by the action of serum-derived proteases during the protein expression in mammalian cells, we prepared engineered HGF where the cleavage site of wild-type HGF (KQLR/V) was mutated to the recognition sequence of Factor Xa (IEGR/V). Similar engineered HGF was previously reported by introducing the Genenase I cleavage site^18^, but we chose Factor Xa to achieve more controlled and efficient cleavage. This mutant HGF (called HGF(Xa) hereafter) would remain single chain (i.e., scHGF(Xa)) during the biosynthesis and purification but can be converted to an active two-chain species (i.e., tcHGF(Xa)) after the treatment with Factor Xa, exposing a new N-terminus at Val495 (Fig. 1A and Supplementary Figure S1).

Recombinant scHGF(Xa) was expressed as a C-terminally His-tagged proteins (Supplementary Figure S1) using the Expi293F cell expression system. After the purification using the Ni-NTA resin and the tag-removal by TEV protease, the scHGF(Xa) was treated with Factor Xa to produce tcHGF(Xa). Both scHGF(Xa) and tcHGF(Xa) were further purified on a heparin column to remove Factor Xa and/or TEV proteases, and showed expected behavior on the reducing and nonreducing SDS-PAGE (Supplementary Figure S2). In order to confirm successful conversion of the scHGF to tcHGF, we assessed the biological activity of both proteins using a Met activation assay in EHMES-1 cell by detecting the phosphorylation of Tyr1234/1235 residues (Fig. 1B). A dose-dependent activation of Met was observed with tcHGF(Xa) as well as with the wild-type HGF (which had been converted to the active two-chain molecule during the biosynthesis), while scHGF(Xa) had minimum effect on the Met phosphorylation. A bell-shaped curve with a decline of phosphorylation level at higher concentration was obtained for both active species, which is consistent with the previous report^12^. The specific activity of the tcHGF(Xa) was ~2-fold higher than the wild-type HGF, which could be due to the different expression hosts and purification procedures. These results show that we could successfully prepare both inactive scHGF(Xa) and active tcHGF(Xa), originated from the same construct.

As we wanted to focus on antibodies that recognize structural features affected by the cleavage between K4 and SP of HGF, we decided to use a truncated HGF protein encompassing the domains K2 to SP (called K2SP hereafter) as an immunogen (Supplementary Figure S1). Both scK2SP(Xa) and tcK2SP(Xa) were prepared by the same method employed for the full-length scHGF(Xa) and tcHGF(Xa) (Supplementary Figure S3), and each protein was independently immunized to MRL/lpr mice. Hybridoma supernatants were sequentially screened using ELISA to obtain candidate clones. As the first screening we employed a direct ELISA, in which each antigen was directly coated onto the microtiter wells. Thirty-seven clones from the mouse immunized with scK2SP(Xa) and 15 clones from the tcK2SP(Xa)-immunized mouse were obtained from this screening. In order to increase the chance of selecting antibodies against native antigen with high affinity, we established a sandwich ELISA system. To this end, full-length HGF (either wild type or Xa version) was C-terminally tagged with a PA tag (Supplementary Figure S1) and displayed on the bottom of a microtiter wells via an anti-PA high affinity antibody NZ-1^19^, reducing the risk of antigen denaturation caused by nonspecific adsorption onto a plastic surface. When the supernatants from the 52 hybridoma clones that were positive in the initial screening were evaluated using this sandwich ELISA, 6 clones (t1E4, t3H3, t5A11, t7E3, t8E4 and t8G12) derived from the mouse immunized with tcK2SP(Xa) were proven positive, while no positive clones were obtained from the mouse immunized with scK2SP(Xa). We speculate that the 37 clones that showed reactivity toward the scK2SP(Xa) in the first screening were either unreactive with native antigen or recognize special epitope that is exposed only in the absence of NK1 region.

**Figure 2:**
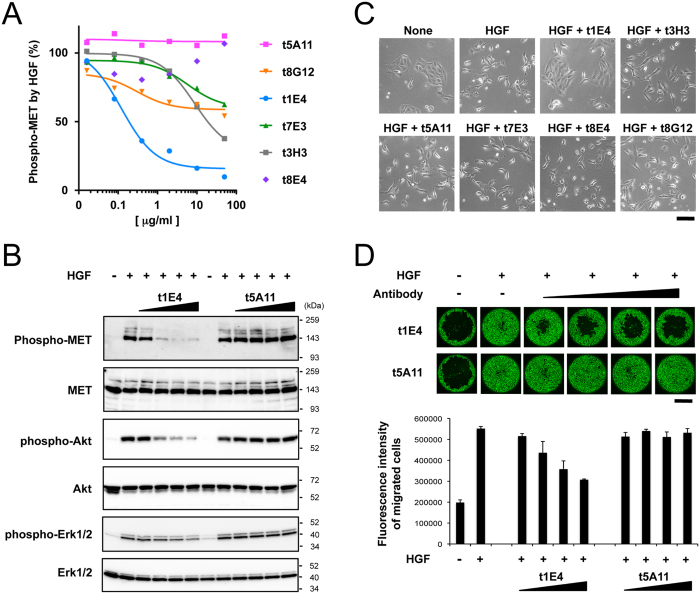
Effect of anti-HGF antibodies on the HGF-induced cellular responses. (**A**) Effect of anti-HGF antibodies on the HGF-induced cellular Met activation. The EHMES-1 cells were stimulated with 20 ng/ml HGF in the presence of vairious antibodies at indicated concentrations for 10 min. The phospho-Met level was determined as in Fig. 1 and expressed as the relative value obtained in the absence of antibody. Data are from a representative experiment in which triplicate determinations were made. (**B**) Inhibition of HGF-induced phosphorylation of Met, Akt, and ERK by t1E4. The EHMES-1 cells were stimulated for 10 min with (+) or without (−) 20 ng/ml HGF, together with the increasing concentrations (0.08, 0.4, 2, and 10 μg/ml) of t1E4 or t5A11 IgGs. Cell lysates were analyzed by SDS-PAGE and Western blotting. (**C**) Effect of anti-HGF antibodies on the ability of HGF to induce MDCK cell scattering. MDCK cells were left untreated (None) or stimulated with 2 ng/ml HGF for 16 h, in the presence of indicated antibodies at 10 μg/ml. Scale bar, 100 μm. (**D**) Inhibition of the HGF-stimulated migration of HuCCT1 human liver bile duct carcinoma cells by t1E4. Images of migrated HuCCT1 cells stimulated with (+) or without (−) 20 ng/ml HGF in the presence of increasing concentrations (0.08, 0.4, 2, and 10 μg/ml) of t1E4 or t5A11 for 24 hrs (upper panels). Cells were stained by calcein-AM. Scale bar, 1 mm. Migrated cells stained with calcein-AM were quantified by fluorescence intensity (lower graph). Data are from a representative experiment in which triplicate determinations were made.

As all isolated antibodies could recognize active form (i.e., two chain) of HGF, we next investigated whether each antibody has any effect on the HGF-Met signaling. Antibodies were added at various concentrations to EHMES-1 cells during the Met activation by a saturating concentration of HGF. As shown in Fig. 2A, Met activation was strongly inhibited by the antibody t1E4, with an IC_50_ value of ~0.15 μg/ml or 0.1 nM. Three other antibodies (t3H3, t7E3 and t8G12) also inhibited Met activation in a dose-dependent manner, but at much weaker level than t1E4. In contrast, t5A11 had no effect even at very high concentration. The effect of t8E4 was unique among 6 since it weakly inhibits the HGF activity only at low concentration range. To validate the authenticity of the Met signaling blockade by t1E4, its effect on the phosphorylation levels of each signaling component was evaluated. As shown in Fig. 2B, addition of t1E4 antibody caused dose dependent reduction of the HGF-induced phosphorylation levels of Met (Tyr1234/1235), Akt (Ser473), and ERK (Thr202 and Tyr204), while none of the phosphorylation events were affected by the addition of t5A11. The inhibitory action of t1E4 was further evaluated by two cell-based assays. First, we tested the effect of all antibodies on the HGF-induced scattering of Madin-Darby canine kidney (MDCK) cells, and confirmed that only t1E4 can strongly suppress the scattering behavior of the cells when added at 10 μg/ml (Fig. 2C). Furthermore, t1E4 antibody, but not t5A11, was able to inhibit the HGF-stimulated migration of human liver bile duct carcinoma cells with a similar concentration dependency as the inhibition of Met phosphorylation in EHMES-1 cells (Fig. 2D). Therefore, we conclude that t1E4 can functionally inhibit HGF-induced Met activation in cells at a concentration range comparable to that of AMG102 (Rilotumumab), an anti-HGF neutralizing antibody that advanced to a phase II trial^20^.

**Figure 3:**
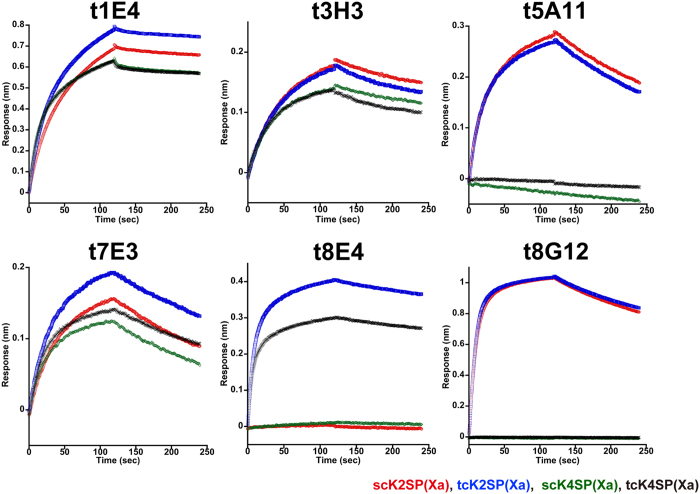
Differential binding of each antibody toward various HGF proteins. Six antibodies (t1E4, t3H3, t5A11, t7E3, t8E4 and t8G12) were separately immobilized onto the Octet sensorchip and binding toward four truncated HGF proteins (red, scK2SP(Xa); blue, tcK2SP(Xa); green, scK4SP(Xa); black, tcK4SP(Xa)) was evaluated. Shown are binding curves after subtraction of the curve obtained with a control IgG. Data are from a representative experiment out of three individual runs.

**Figure 4:**
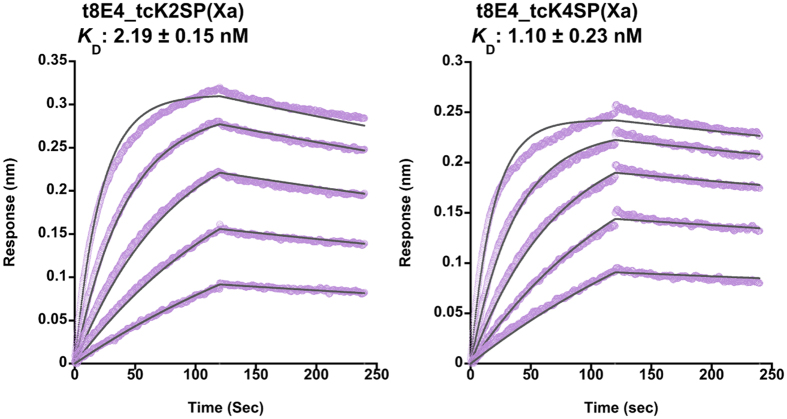
Determination of the binding affinities of t8E4 toward different truncated tcHGF proteins. The sensorchip immobilized with the t8E4 antibody was incubated with five different concentrations (6.25, 12.5, 25, 50, and 100 nM) of tcK2SP(Xa) or tcK4SP(Xa) analytes. Actual binding curves (lavender) are overlaid with the fitting curves (thin black lines) used to derive the *K*_D_ values. Experiments were repeated three times and the *K*_D_ values are expressed as mean ± SD (n = 3) at the top.

**Figure 5:**
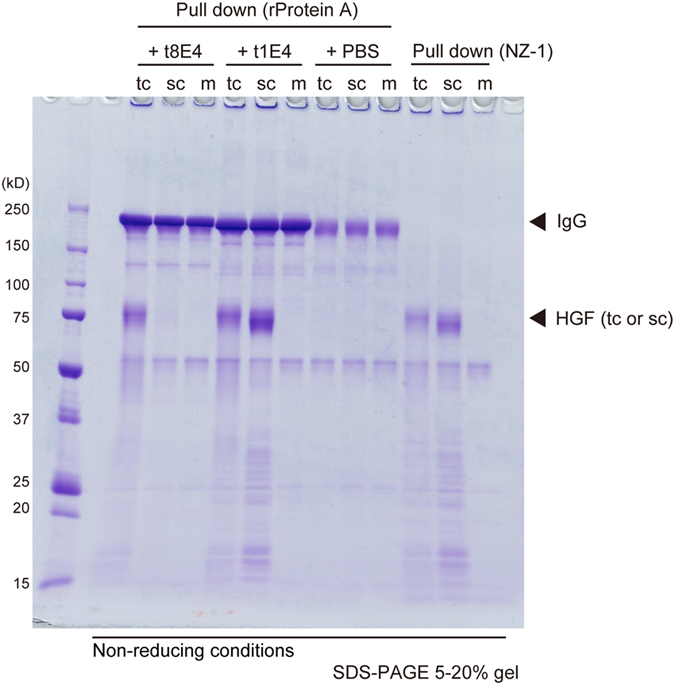
Recognition of the wild-type two-chain HGF by the t8E4 antibody. The culture media from tcHGF-PA (tc), scHGF(Xa)-PA (sc), or mock (m)-transfected HEK293T cells were immunoprecipitated with either t8E4, t1E4, or NZ-1, followed by the SDS-PAGE and Coomassie staining. The wild-type HGF protein was fully converted into two-chain species because of the cleavage by serum-derived protease during the expression, while the engineered “Xa” version remained as single chain species under the same condition. Because of the non-reducing condition employed here, however, both proteins migrated as an ~75-kDa band (arrowhead).

In order to map the location of the epitope for the 6 antibodies, binding toward various HGF fragments were evaluated using an Octet system. The fragments tested included the K2SP(Xa) construct used in the immunization and a shorter version lacking the K2 and K3 (called K4SP hereafter). They were either untreated (single chain) or treated with Factor Xa (two chain), resulting in 4 different samples, namely, scK2SP(Xa), tcK2SP(Xa), scK4SP(Xa) and tcK4SP(Xa) (Supplementary Figure S1). As shown in Fig. 3, antibodies t1E4, t3H3 and t7E3 bound all fragments nearly equally, indicating that they can bind not only the two chain but also the single chain HGF and each recognizes an epitope located within the K4-SP region. In contrast, t5A11 and t8G12 bound scK2SP(Xa) and tcK2SP(Xa) while they showed no binding toward scK4SP(Xa) and tcK4SP(Xa). Therefore, epitopes for these two antibodies are located primarily in the K2-K3 region of HGF, which are equally exposed on both tcHGF and scHGF. Intriguingly, the binding specificity of t8E4 was totally different from these 5 antibodies; it bound both K2SP and K4SP fragments when they are in the two-chain state but showed no reactivity toward the single chain versions. We determined the binding affinity of t8E4 toward the HGF proteins by varying the analyte concentration in the Octet experiments (Fig. 4), and obtained K_D_ values of 2.19 ± 0.15 nM and 1.10 ± 0.23 nM (mean ± SD, n = 3) for tcK2SP(Xa) and tcK4SP(Xa), respectively. These results strongly suggest that t8E4 recognizes an epitope present in the K4-SP region, and that this site is formed only after the cleavage between Arg494 and Val495 occurs. The specificity toward cleaved HGF was also checked by an immunoprecipitation assay using wild-type HGF devoid of the engineered factor Xa site. As shown in Fig. 5, t8E4 can specifically immunoprecipitate tcHGF but not scHGF, while t1E4 and anti-tag antibody (NZ-1) precipitate both scHGF and tcHGF equally well. This clearly demonstrates that t8E4 recognizes natural epitope present in the active tcHGF, rather than an artificial epitope involving the engineered factor Xa site. In summary, we succeeded in generating 6 antibodies that react with native human HGF, including one antibody (t1E4) with function-neutralizing activity and one (t8E4) with an ability to distinguish active tcHGF from inactive scHGF.

Although numerous anti-HGF antibodies have been developed and reported^21^, detailed biochemical characterization of each antibody such as determining the epitope location and recognition specificity are generally scarce. Particularly, we could find only one antibody (AMG102) that was experimentally shown to distinguish tcHGF from scHGF through our literature search^20^. In their paper, Burgess *et al*. reported that, using an immunoprecipitation experiment, AMG102 preferentially bound mature HGF (tcHGF) from the mixture of mature HGF and the non-cleaved pro-HGF (scHGF). However, they also found that this distinction was not perfect, because AMG102 did immunoprecipitate scHGF at higher concentration. In contrast, the tcHGF-specific antibody we report here (t8E4) does not bind uncleaved scHGF at all, suggesting that HGF cleavage is absolutely required for the formation of its epitope structure. In fact, there is an essential difference between AMG102 and t8E4 in that the former has strong inhibitory activity on the Met activation by the HGF, while the latter shows marginal inhibitory effect (Fig. 2A). Whereas AMG102 was predicted to compete with Met upon its binding to HGF SP domain^20^, we found that t8E4 does not directly interfere with the Met-HGF interaction (data not shown). Therefore, t8E4 seems to report a genuine conformational change(s) within the HGF protein that constitute an essential step during the HGF “activation”. Further analysis of the t8E4 epitope structure in detail may help solving the long-standing question as to the structural mechanism of HGF activation.

Finally, a panel of monoclonal antibodies we report here may prove useful in facilitating the structural determination of HGF. Antibodies and their fragments are often used as “crystallization chaperone”, where the likelihood of obtaining well-ordered crystals of flexible proteins are increased by the formation of complex with a binder^22^. Currently, the structural information of HGF is available for only a limited set of domains including NK1, NK2^23^, and SP^10^,^11^,^24^, and no structures are available for K3 and K4 domains. More importantly, there is no high resolution structures available for the full-length HGF either before or after the proteolytic activation. Although the crystal structure of full-length plasminogen is available^25^, it does not serve as a good template for a homology modeling of full-length scHGF due to the presence of an additional kringle domain. We attempted to crystallize full-length tcHGF or scHGF in many conditions with no success, making us to speculate that the intrinsic mobile nature of the full-length HGF may hamper the efficient crystallization. In this regard, the antibodies reported here may rigidify the HGF and its fragments upon the complex formation and accelerate the crystallization, ultimately helping us to understand the mechanism of HGF-Met signaling.

## Materials and Methods

### DNA construction

Full-length human HGF cDNA coding for variant 3 (NM_001010932.2)^26^ was used in all plasmid constructions throughout the manuscript, while the residue numbering was based on the sequence of variant 1 which contained additional 5 amino acids in the K1 domain. For construction of full-length HGF, the entire coding sequence was appended with a hexahistidine tag or a PA tag^19^ at the C-terminus. For construction of the “Xa-cleavable” version, four-residue segment preceding the processing site Val495 was changed from KQLR to IEGR by using extention PCR method. To prepare N-terminally truncated constructs, segments corresponding to residues Glu183-Ser728 (for K2SP) or Gly388-Ser728 (for K4SP) were PCR-amplified and cloned into a pcDNA3.1-based vector containing a prolactin signal sequence and a hexahistidine tag. All tag sequences were preceded by a tobacco etch virus (TEV) protease cleavage site. Some of the constructs contained additional point mutations to eliminate N-linked glycosylation sites (N294Q, N402Q, T476G, N566Q, and N653Q) or an unpaired cysteine (C561S), as denoted in Supplementary Figure S1.

### Protein expression and purification

For the expression of C-terminally PA-tagged HGF proteins, vectors coding for tcHGF-PA or scHGF(Xa)-PA were transfected into HEK293T cells using X-tremeGENE HP DNA Transfection Reagent (Roche) and cultured in a serum-containing medium. The culture media were collected after 3–4 days and used for the sandwitch ELISA or the pull-down assay. For the purification of C-terminally His-tagged HGF proteins, plasmid vectors were used to transfect Expi293F cells (ThermoFisher scientific) according to the manufactor’s protocol. During the expression of full-length glycosylated HGF, 5 μM kifunensine (Cayman chemical) was added to produce recombinant proteins with a homogeneous glycoform. The culture media were collected 3 days post transfection, and the proteins were purified on an Ni-NTA agarose column (Qiagen). After the removal of the C-terminal His-tag by an overnight incubation with the TEV protease (0.1 mg/ml) at room temperature, the scHGF(Xa) proteins were further purified on a Hitrap Heparin HP column (GE Healthcare) and dialysed against 20 mM Tris-HCl, pH 7.5 and 300 mM NaCl to obtain purified scHGF(Xa). To prepare tcHGF(Xa), the TEV-treated samples above were further incubated overnight at 20 °C with 6 μg/ml Factor Xa (Novagen) and purified as in the case of scHGF(Xa). For the purification of protein samples used in the Octet experiments, an additional gel filtration step on a Superdex 75 10/300 GL column (GE Healthcare) equilibrated in 20 mM Tris-HCl, pH 7.5 and 150 mM NaCl (TBS), was included.

### Cell-based Met signaling assays

Cellular Met activation by HGF was evaluated by using EHMES-1 human mesothelioma cells. For the quantitation of the overall Met-phosphorylation, EHMES-1 cells were seeded at 2 × 10^4^ cells per well in a 96-well black μClear-plate (Greiner Bio-One) and cultured for 24 h. The cells were stimulated with HGF, tcHGF(Xa), or scHGF(Xa) in RPMI1640 medium supplemented with 10% fetal bovine serum (FBS) for 10 min, in the presence of various concentrations of anti-HGF antibodies. After washing with ice-cold phosphate-buffered saline (PBS), cells were fixed with 4% paraformaldehyde in PBS for 30 min, washed three times with PBS, followed by blocking with 5% goat serum, 0.02% Triton X-100 in PBS for 30 min and incubation with anti-phospho-Met (Tyr1234/1235) XP rabbit monoclonal antibody (Cell Signalinf Technologies, D26) diluted at 1:1,000 with PBS containing 1% goat serum for 12 h at 4 °C. The cells were washed three times with PBS and incubated for 1 h with horseradish peroxidase (HRP)-conjugated anti-rabbit goat antibody diluted at 1:1,000 with PBS containing 1% goat serum, then washed four times with PBS. Chemiluminescence was developed with ImmunoStar LD reagent (Wako) and measured by an ARVO MX plate reader (Perkin Elmer). Relative Met phosphorylation was calculated as (Chemiluminescence unit of sample – chemiluminescence unit of mock control)/(chemiluminescence unit of 1.3 nM hHGF – chemiluminescence unit of mock control). For Western blotting, EHMES-1 cells were cultured in a 12-well plate until they were 80–90% confluent, subjected to a serum starvation for 6 h, and were stimulated with or without 20 ng/ml HGF in the presence of varying concentrations of purified anti-HGF antibodies t1E4 and t5A11 for 10 min. The wells were washed twice with ice-cold PBS, and the cells were lysed by adding 150 μl of SDS-PAGE sample buffer followed by sonication and clarification by centrifugation. The 20 μl of lysates were subjected to SDS-PAGE using a 3–10% (for Met and Akt) or a 5–20% (for Erk) gradient polyacrylamide gel. Upon the transfer onto the PVDF membrane, separated cellular proteins were probed with the following primary antibodies: anti-phospho-Met (Y1234/1235) (Cell Signaling Technology, D26), anti-Met (Cell Signaling Techonology, 25H2), anti-phospho-Akt (S473) (Cell Signaling Technology, D9E), anti-Akt (Cell Signaling Technology), anti-phospho-ERK1/2 (T202/Y204)(Cell Signaling Technology, D13.14.4E), and anti-ERK1/2 (Cell Signaling Technology, 137F5). The bound antibodies were visualized either by HRP-conjugated anti-rabbit immunoglobulins or anti-mouse immunoglobulins (Dako), followed by the development with the ImmunoStar LD reagent (Wako). For cell scatter assay, Madin-Darby canine kidney (MDCK) cells were seeded at 2 × 10^4^ cells per well in a 12-well plate in DME medium supplemented with 10% FBS, and incubated with or without 2 ng/ml HGF plus 10 μg/ml anti-HGF antibodies. After 16 h, the phase contrast microscopic images of the cells were recorded. Cell migration activity was evaluated by using Oris Cell Migration Assay Kit (Platypus Technologies). Briefly, HuCCT1 human liver bile duct carcinoma cells were seeded at 4 × 10^4^ cells per well in a 96-well plate and cultured for 24 h in RPMI medium with 10% FBS. The stoppers were removed and the cells were washed twice and cultured in FBS-free RPMI medium with or without 20 ng/ml HGF to allow the migration in the presence of varying concentrations of anti-HGF antibodies t1E4 and t5A11. After culturing for 24 h, adherent cells were stained with 5 μg/ml calcein-AM for 15 min and the migration was quantitated by ARVO MX fluorescence plate reader. The cell images were also recorded by a fluorescence microscope BIOREVO BZ-9000 (KEYENCE).

### Hybridoma production

Two MRL/lpr mice were purchased from SLC Japan (Shizuoka, Japan), and were housed under pathogen-free conditions. The Animal Care and Use Committee of Tohoku University approved the animal experiments described herein. All experiments were performed in accordance with animal guidelines and regulations determined by Tohoku University. Two MRL/lpr mice (SLC Japan, Shizuoka, Japan) were independently immunized by an intraperitoneal (i.p.) injection of 100 μg of scK2SP(Xa) or tcK2SP(Xa) together with Imject Alum (Thermo Fisher Scientific Inc., Waltham, MA). After several additional immunizations, a booster injection was given i.p. two days before the harvest of spleen cells. The spleen cells were fused with P3U1 cells using PEG1500 (Roche Diagnostics, Indianapolis, IN). The fused cells were grown in a RPMI1640 medium supplemented with hypoxanthine, aminopterin, and thymidine selection medium supplement (Thermo Fisher Scientific Inc.). For the direct ELISA, recombinant proteins (scK2SP(Xa) or tcK2SP(Xa)) were immobilized on Nunc Maxisorp 96-well immunoplates (Thermo Fisher Scientific Inc.) at a concentration of 2 μg/ml for 30 min. After blocking with PBS containing 1% BSA, the plates were incubated with hybridoma culture supernatants, probed with a HRP-conjugated anti-mouse IgG (Dako; Agilent Technologies, Inc., Glostrup, Denmark, diluted at 1:3,000), followed by a development with a 1-Step Ultra TMB-ELISA (Thermo Fisher Scientific Inc.) and the measurement of the optical density at 655 nm using an iMark microplate reader (Bio-Rad Laboratories Inc., Hercules, CA). For the sandwich ELISA, the rat anti-PA tag antibody NZ-1 (Wako Pure Chemical Industries, Ltd.) was immobilized on Nunc 96-well immunoplates at a concentration of 10 μg/ml for about 3 h at room temperature. After blocking for overnight with the Blocking One solution (Nacalai Tesque, Inc.), the conditioned media from HEK293T cells expressing C-terminal PA tagged HGF proteins (tcHGF-PA and scHGF(Xa)-PA) or the conditioned medium from the mock-transfected HEK293T were added and incubated for 1 h at room temperature, followed by washing with TBS. Then, the plates were incubated with the culture supernatant form hybridoma clones for 1 h at room temperature and washed with TBS, followed by an addition of HRP-conjugated goat anti-mouse IgG (preadsorbed with rat IgG, SouthernBiotech, Inc.) diluted at 1:3,000. The enzyme reaction was developed with ABTS microwell peroxidese substrate (KPL, Inc.) and the optical density was measured at 405 nm using a SH-9000 microplate reader (Corona electric, Inc.). Single cell cloning of hybridoma was performed by limiting dilution. The cloned hybridoma cells were initially cultured in a RPMI1640 medium supplemented with 10% FBS, 1% non-essential amino acids, 0.5% penicillin-streptomycin and 1% Na-pyruvate, with the addition of Briclone (NICB) if needed. The hybridoma cells were gradually adapted to serum free media (HyClone SFM4Trnasfx293,Thermo Fisher Scientific Inc). The antibodies from the culture media of hybridoma cells were purified using the recombinant protein A-Sepharose (GE Healthcare). The subclasses of each antibody are: [t1E4 (IgG2b, kappa), t3H3 (IgG1, kappa), t5A11 (IgG1, kappa), t7E3 (IgG1, kappa), t8E4 (IgG1, kappa) and t8G12 (IgG2b, kappa)].

### Octet experiment

Octet RED 96 system (ForteBio) was used for the measurement of bindings between the antibodies and the truncated HGF proteins. Purified antibodies (20 μg/ml) diluted in 10 mM sodium acetate, pH 6.0, were immobilized onto AR2G biosensors (ForteBio) using the amine coupling chemistry. The binding experiments were performed in TBS containing 0.1% BSA and 0.02% Tween-20 at 25 °C. After each cycle of binding experiment, antibody-immobilized biosensors were regenerated by dipping in a regeneration buffer (10 mM glycine-HCl, pH 3.0). The K_D_ values were determined using Octet Data analysis software 7.1 (ForteBio) using a 1:1 global fitting model.

### Pull-down assay

One-milliliter culture media from the HEK293T cells transfected with tcHGF-PA, scHGF(Xa)-PA or mock vector were incubated with 40 μl of rProtein A sepharose (GE Healthcare) in the presence or absense of 10 μg of t8E4 or t1E4 antibodies for 2 h at 4 °C. Each culture medium was separately incubated with 40 μl of NZ-1(anti-PA tag)-immobilized Sepharose to evaluate the amount of total PA-tagged proteins present in the media. The beads were washed twice with 1 ml of TBS and the bound proteins were eluted with SDS-containing sample buffer and analysed by a SDS-PAGE. Three individual pull-down experiments were performed to confirm the results, with a representative one shown in Fig. 5.

## Additional Information

**How to cite this article**: Umitsu, M. *et al*. Probing conformational and functional states of human hepatocyte growth factor by a panel of monoclonal antibodies. *Sci. Rep.* **6**, 33149; doi: 10.1038/srep33149 (2016).

## Supplementary Material

Supplementary Information
