# Supplementary material for: Probing conformational and functional states of human hepatocyte growth factor by a panel of monoclonal antibodies
Source: Sci Rep. 2016 Sep 9;6:33149. doi: 10.1038/srep33149 (PMC5017023; doi:10.1038/srep33149)
Supplement: Supplementary Information [file srep33149-s1.pdf]

Supplementary Information

Title

Probing conformational and functional states of human hepatocyte growth factor by a panel of monoclonal antibodies

Authors

Masataka Umitsu, Katsuya Sakai, Satoshi Ogasawara, Mika K. Kaneko, Ryoko Asaki, Keiko Tamura-Kawakami, Yukinari Kato, Kunio Matsumoto, Junichi Takagi

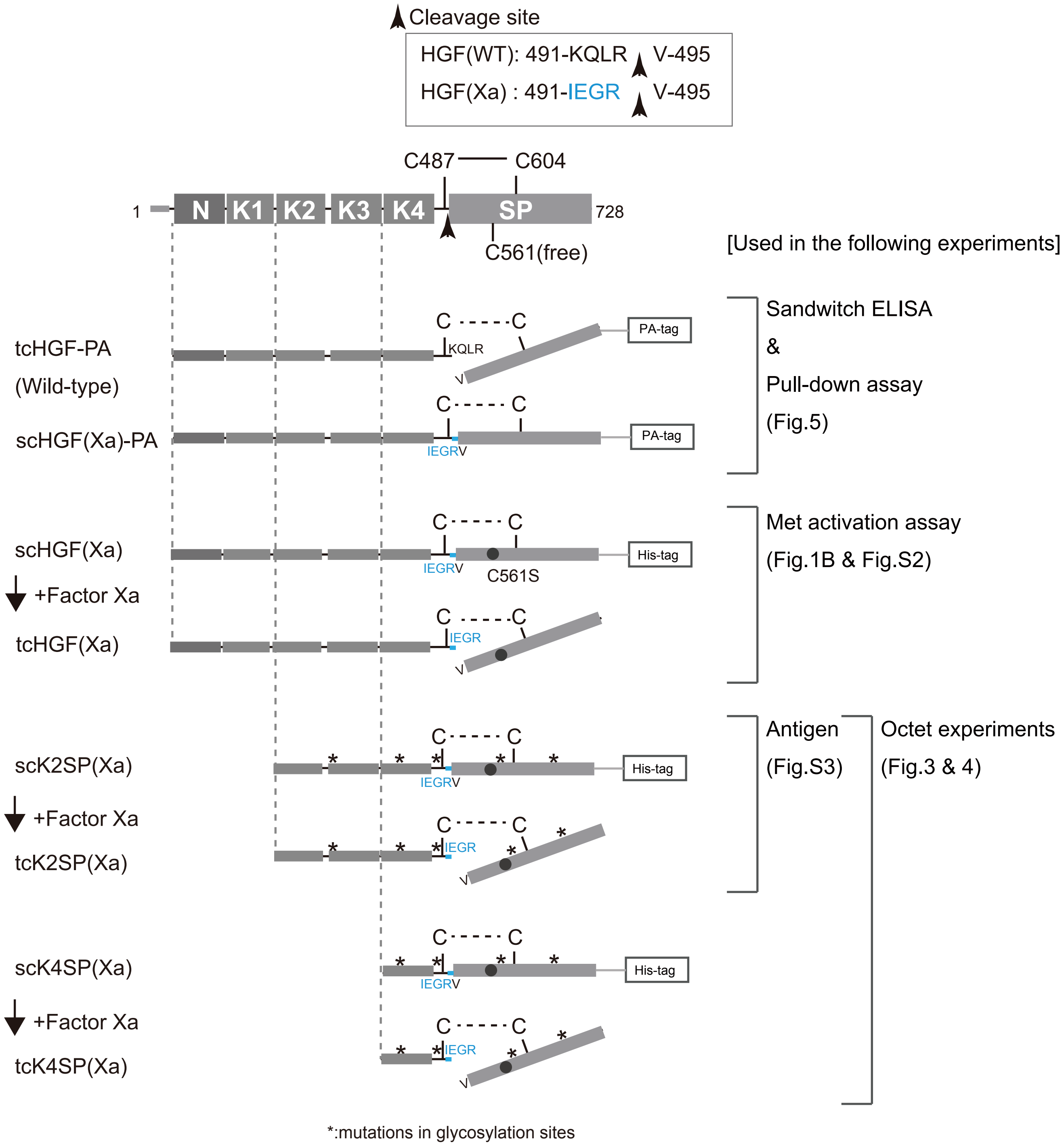

Figure S1_Umitsu

Supplementary Figure S1

Figure S1. The HGF protein constructs used in this study. Domain organization and various sequence features for each construct are shown as schematic representations. Constructs were differently tagged at the C-terminal and contained either wild-type (black) or engineered (blue) sequence at the cleavage site. Some constructs contained the Asn to Gln mutations at the N-glycosylation sites (asterisks) and/or C561S mutation (black dot). To facilitate the understanding of the experimental settings, figure numbers and experimental steps that used each construct were indicated in the right.

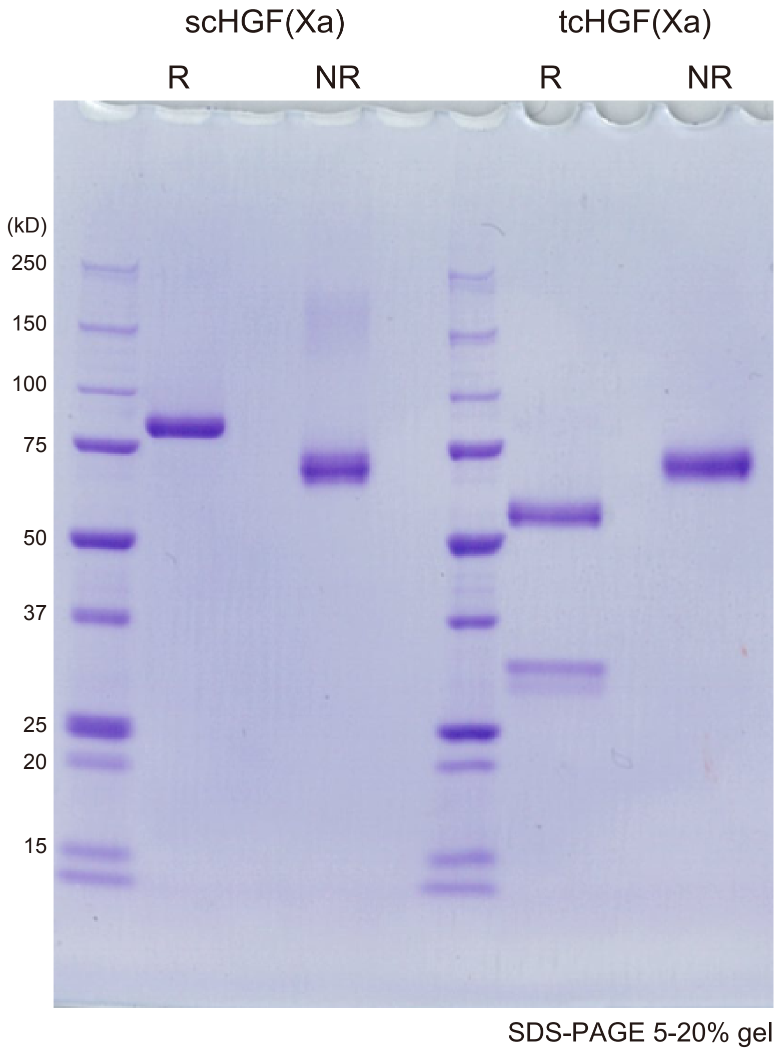

Figure S2_Umitsu

Supplementary Figure S2

Figure S2. SDS-PAGE analysis of the purified scHGF(Xa) and tcHGF(Xa) proteins. The samples were run under non-reduding (NR) or reducing (R) conditions. Note that both samples show single band with a similar mobility (~70 kDa) under nonreducing condition, while in the reducing condition tcHGF(Xa) migrates as two bands corresponding to the α- (~58 kDa) and the β- (~30 kDa) chains.

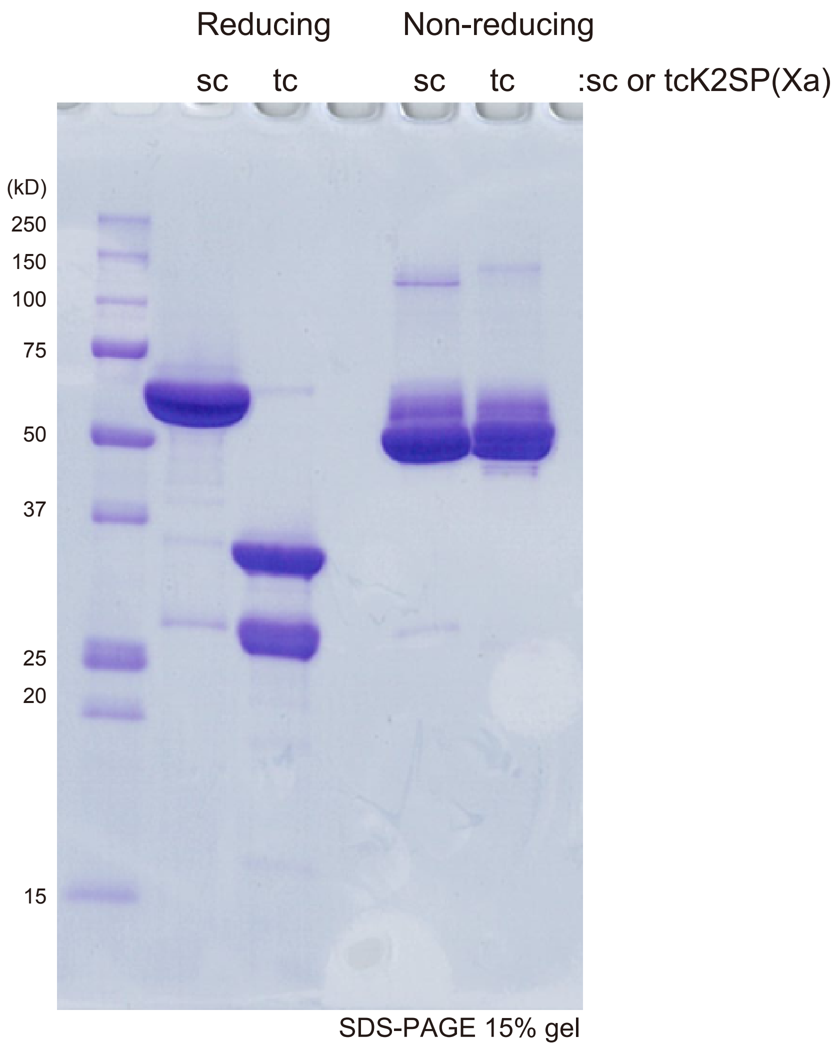

Figure S3_Umitsu

Supplementary Figure S3

Figure S3. SDS-PAGE analysis of the purified scK2SP(Xa) and tcK2SP(Xa) proteins. The samples were run under non-reduding or reducing conditions. Both samples show single band with a similar mobility (~50 kDa) under nonreducing condition, which is splited into two bands under the reducing condition for the tcHGF(Xa) but not the scHGF(Xa). Note the near-absence of the tc species in the scK2SP(Xa) sample and the sc species in the tcK2SP(Xa) sample.
